# Efficacy of high dose methotrexate in pediatric auto-immune uveitis

**DOI:** 10.1186/1546-0096-12-S1-P234

**Published:** 2014-09-17

**Authors:** Wineke Armbrust, Wietse Wieringa, Leonie Los

**Affiliations:** 1 Beatrix children's hospital, Groningen, Netherlands; 2 Ophthalmology, University Medical Center Groningen, Groningen, Netherlands

## Introduction

Efficacy of high dose methotrexate in pediatric auto-immune uveitis.

## Objectives

To compare the efficacy of high dose (≥15mg/m^2^/week ) Methotrexate (MTX) versus low dose (<15mg/m^2^/week) MTX in relation to time to disease remission.

## Methods

Retrospective analysis of 46 pediatric patients with uveitis with or without underlying systemic disease treated with MTX at the University Medical Center Groningen (The Netherlands ) between 1993 and 2013. The SUN (Standardization of Uveitis Nomenclature) workinggroup criteria were used for endpoints. Other endpoints included visual outcome, steroid-sparing effect, cumulative dose MTX to disease remission and side effects.

## Results

Mean age at onset of uveitis was 6.6 years (1.7 – 18). Male:female ratio was 24/22. In 36 patients, bilateral disease was found. Most patients (n=27) had anterior uveitis, followed by intermediate (n=9), pan (n=8) and posterior uveitis (n=2). Ocular complications related to the uveitis were found in 36 patients. Cataract surgery was performed in 28 patients and glaucoma surgery in 20 patients. JIA was the underlying systemic disease in 23 patients. One patient was diagnosed with Cogan’s syndrome, in 22 patients no underlying systemic disease was found. ANA positivity was found in 24 out of 46 patients. In 40 patients, MTX use had been sufficiently long for analysis. In 28 of these patients, disease remission was achieved in ( median) 26.7 (range 2.5- 146) months. Patients treated with a lower maximum dose of MTX had a longer time to disease remission (median 26.1, range 2.8 – 147.1 months) than patients treated with a higher dose of MTX (median 19.7, range 2.5 – 29.8 months) (p-value 0.02). No statistical significant differences were found in steroid-sparing effect, cumulative dose MTX to disease remission and side effects.

## Conclusion

In this retrospective study on pediatric auto-immune uveitis, high dose MTX seems to result in a quicker disease remission.

## Disclosure of interest

None declared.

